# Clinical benefit for clinical sequencing using cancer panel testing

**DOI:** 10.1371/journal.pone.0247090

**Published:** 2021-02-26

**Authors:** Sadaaki Nishimura, Atsushi Sugimoto, Shuhei Kushiyama, Shingo Togano, Kenji Kuroda, Yurie Yamamoto, Makoto Yamauchi, Toshiyuki Sumi, Hiroyasu Kaneda, Tomoya Kawaguchi, Minoru Kato, Mizuki Tagami, Naoto Oebisu, Manabu Hoshi, Kenjiro Kimura, Shoji Kubo, Kazuya Muguruma, Tsutomu Takashima, Masaichi Ohira, Masakazu Yashiro

**Affiliations:** 1 Department of Gastroenterological Surgery, Osaka City University Graduate School of Medicine, Osaka, Japan; 2 Molecular Oncology and Therapeutics, Osaka City University Graduate School of Medicine, Osaka, Japan; 3 Cancer Center for Translational Research, Osaka City University Graduate School of Medicine, Osaka, Japan; 4 Department of Hepato-Biliary-Pancreatic Surgery, Osaka City University Graduate School of Medicine, Osaka, Japan; 5 Department of Obstetrics and Gynecology, Osaka City University Graduate School of Medicine, Osaka, Japan; 6 Cancer Genomic Center, Osaka City University Graduate School of Medicine, Osaka, Japan; 7 Department of Clinical Oncology, Osaka City University Graduate School of Medicine, Osaka, Japan; 8 Department of Respiratory Medicine, Osaka City University Graduate School of Medicine, Osaka, Japan; 9 Department of Urology, Osaka City University Graduate School of Medicine, Osaka, Japan; 10 Department of Ophthalmology and Visual Sciences, Osaka City University Graduate School of Medicine, Osaka, Japan; 11 Department of Orthopedic Surgery, Osaka City University Graduate School of Medicine, Osaka, Japan; 12 Department of Breast and Endocrine Surgery, Osaka City University Graduate School of Medicine, Osaka, Japan; CNR, ITALY

## Abstract

**Background:**

Clinical sequencing using a panel of genes has recently been applied worldwide for patients with refractory solid tumors, but the significance of clinical sequencing using gene panel testing remains uncertain. Here we sought to clarify the feasibility and utility of clinical sequencing in the treatment of refractory tumors at our hospital.

**Methods:**

A total of 39 patients with advanced solid tumors treated at our hospital between 2018 and 2020 were enrolled in the clinical sequencing. Among them, we identified 36 patients whose tissue samples were of suitable quality for clinical sequencing, and we analyzed the genomic profiles of these tumors.

**Results:**

Pathogenic alterations were detected in 28 (78%) of the 36 patients. The most common mutation was *TP53* (55%), followed by *KRAS* (22%), and the highest frequency of gene amplification was *ERBB2* (17%). Nine of the 36 patients were identified as candidates for novel molecular-targeted therapy based on their actionable gene alterations, but only one case ended up receiving novel targeted therapy following the genetic tests.

**Conclusions:**

Our current results suggested that clinical sequencing might be useful for the detection of pathogenic alterations and the management of additional cancer treatment. However, molecular target based on actionable genomic alteration does not always bridge to subsequent therapy due to clinical deterioration, refusal for unapproved drug, and complexity of clinical trial access. Both improved optimal timing of clinical sequencing and a consensus about its off-label use might help patients receive greater benefit from clinical sequencing.

## Introduction

Several studies have defined the genomic landscape of cancer with the use of next-generation sequencing (NGS) technology, and they have detected some candidate driver-gene alterations of cancer that allow the tumor cells to survive and spread [1–4]. Clinically, gene panel testing (which uses NGS to target a limited number of cancer-associated genes) has been the most practical genome profiling method worldwide [5–8]. NGS is often used for patients with advanced refractory cancer who fail to respond to standard therapy, and it is linked to a new alternative known as ’clinical sequencing’.

FoundationOne^®^ CDx and MSK-IMPACT, which are comprehensive genome profiling tests for all solid tumors, have been approved by the U.S.FDA and used for the clinical sequencing of over 10,000 patients [9,10]. In Japan, a total of 230 patients with advanced solid tumors underwent clinical sequencing using the NCC Oncopanel as part of the TOP-GEAR project at the National Cancer Center Hospital (UMIN000011141), and the genetic tests revealed that 13% of the patients were candidates for novel targeted therapy [11]. In this context, the use of the NCC Oncopanel as well as the FoundationOne^®^ CDx has been covered under Japan’s national health insurance since June 2019. Thus, there have been few evaluations of the feasibility and utility of clinical sequencing in cancer treatment in Japan [11–13]. At our hospital, clinical sequencing has been introduced for cancer patients as an application of precision oncology. Here we summarize the results of 39 patients who underwent panel testing as clinical sequencing. We also provide the results of our assessment of the feasibility of clinical sequencing using panel testing.

## Material and methods

### Patients

We recruited the patients with refractory cancer who are eligible to underwent gene panel testing at our hospital between 2018 and 2020. For the patients included in this study, the following eligibility was confirmed before initiation of clinical sequencing: [1] refractory to standard treatment for solid tumor or no evidence of standard therapy for primary unknown cancer; [2] evidence of good performance status; [3] evidence of over 16 years old. [4] evidence of leftover specimens for sequencing. Specimens obtained by surgery, core and fine needle biopsy were available. All protocols were conducted after obtaining written informed consent from all patients in accordance with the approved procedures at our hospital.

### Gene panel testing based on NGS

**Fig 1:**
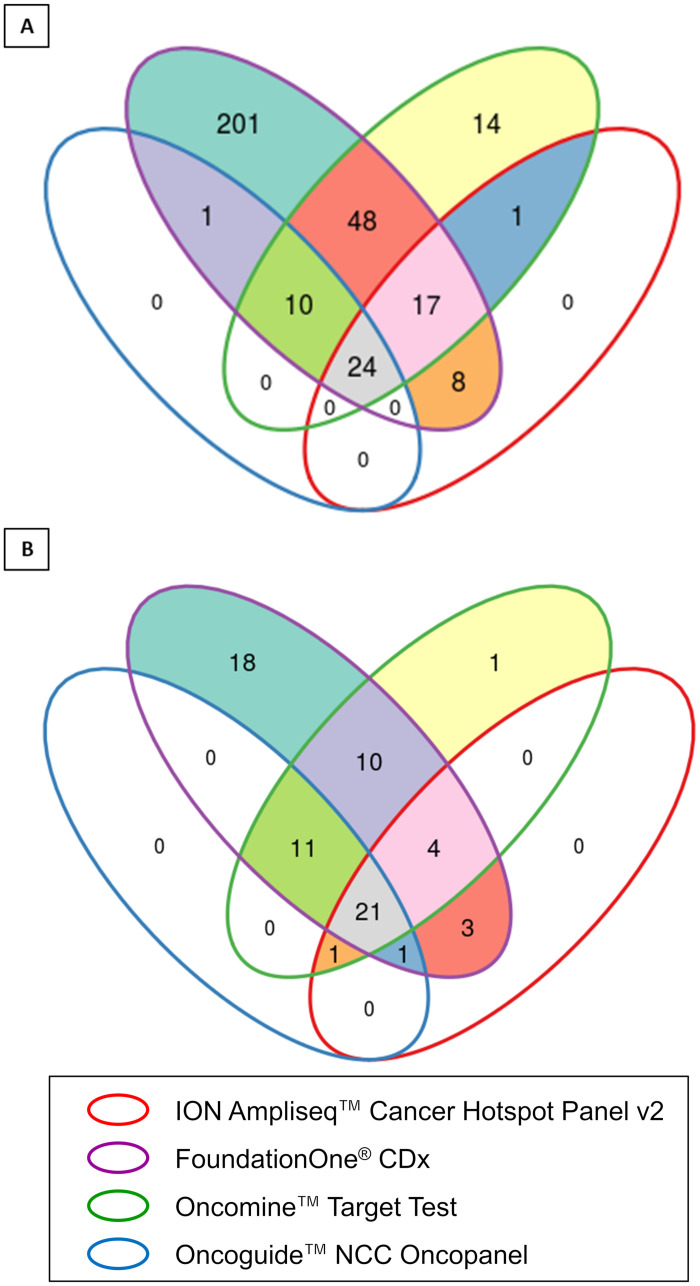
Venn diagram of gene lists overlap by four types of panel testing. **A**, The Venn diagram of the distribution of 324 genes targeted for all exons or hotspot regions in four panels. **B**, The Venn diagram of the distribution of druggable genes listed in four panels.

For current clinical sequencing, four types of gene panel testing were performed in this study, as follows; [1] OncoGuide^™^ NCC Oncopanel (Sysmex, Kobe Japan) targeted the coding exons of 114 cancer associated genes including 12 fusion genes was performed after implement for coverage under national health insurance. To detect somatic mutation, peripheral blood was used as a reference of germline mutation in this sequencing. [2] FoundationOne^®^ CDx (Foundation Medicine, Inc., Cambridge, MA), which detected substitutions, insertion-deletions and copy-number alterations in 309 genes and select 36 gene arrangements, was approved by Japanese regulators, as well as NCC Oncopanel. [3] Oncomine^™^ Target Test (Thermo Fisher Scientific, Waltham, MA), which interrogated prominent mutational hotspots only in 46 genes except for *TP53* and examined 12 gene rearrangements, was underwent as a part of Japan medical care sponsored by Osaka University hospital. Over 20% of tumor cellularity in FFPE sample was available for this panel testing. [4] ION Ampliseq^™^ hotspot panel v2 (Thermo Fisher Scientific, Waltham, MA), which research somatic mutations across the hotspot regions of 50 cancer associated genes, was performed as a part of prospective clinical trial sponsored by Kindai hospital (UMIN000029779). Oncomine^™^ Target Test and ION Ampliseq^™^ hotspot panel v2 were performed as a clinical study approved by Ethical Committee of Osaka City University Graduate School of Medicine, Osaka, Japan (Permission number: 4199, 3925), whereas FoundationOne^®^ CDx and NCC Oncopanel were clinically available. S1–S4 Tables list details of genes in these panels, including actionable genes caused by variant, copy number alteration and rearrangement. Among a total of 324 genes targeted in these panels, 24 genes were confirmed in the intersection of all four panels (Fig 1A), whereas 70 actionable genes for FDA-approved targeted therapies, including genome-matched and related target, was observed in these panels (Fig 1B).

### Criteria for selecting gene panel testing

The type of panel testing was selected according to test date. The Oncomine^™^ Target Test or the ION Ampliseq^™^ hotspot panel v2 were conducted from April 2018 to May 2019 as a part of clinical trial. The NCC Oncopanel and the FoundationOne^®^ CDx were conducted from June 2019 to August 2020. Each panel testing was explicated by expert doctors on the basis of cancer type.

### Tumor mutational burden

Tumor mutational burden (TMB), which is related to response immune checkpoint therapy, was also analyzed in NCC Oncopanel and FoundationOne^®^ CDx [11,14]. TMB was classified as 2 categories, High-TMB and Low-TMB. High-TMB was defined as over 10 mutations/Megabase, otherwise Low-TMB was defined as under 10 mutations/Mb, as previously reported [15].

### Reporting and curation of genetic alteration

Sequencing was performed using NGS system according to each platform. After sequencing with NGS, bioinformatics analysis was performed to call genetic alterations using each NGS data analysis pipeline, which was specified in S1 File. Finally, important filtered variants, which were classified according to standardized guidelines [16], were discussed at the molecular tumor board, called the “expert panel”. Each expert panel was composed of several specialists representing various disciplines, including the attending physician, an oncologist, a doctor in charge of clinical testing, a genetic counselor, a pathologist, a bioinformatician and a doctor specializing in clinical genetics. They notably curated the explanations on genetic alteration according to Clinvar (https://www.ncbi.nlm.nih.gov/clinvar/), COSMIC database (http://cancer.sanger.ac.uk/cosmic) and gnomAD (http://gnomad.broadinstitute.org/). And they collectively interpreted analysis results and provided the attending doctor with proposal for treatment option, as well as the information of cancer diagnosis and prognosis, referring to knowledgebase for clinical interpretation of cancer variants, such as OncoKB (https://www.oncokb.org/), CIVic (https://civicdb.org/home) and Diagnostic guidance (version 1.0) based on gene panel testing using NGS issued jointly by the Japanese Society of Medical Oncology, the Japan Society of Clinical Oncology and the Japanese Cancer Society in detail (https://www.jsmo.or.jp/about/doc/20171011_01.pdf). The classification of evidence level for genetic alteration in Diagnostic guidance (version 1.0) was described in previous report [11]. After expert panel discussion, the curated report based on this council was returned to clinician.

### Statistical analysis

Intersection and overlapping of gene lists between gene panel testing was calculated by Intervene (https://asntech.shinyapps.io/intervene/). Descriptive statistics were used to summarize patient characteristics and genomic profiling using cBioportal [17,18]. Survival rates were estimated using the Kaplan-Meier method and performed using GraphPad Prism 8.3.0 (GraphPad Software, La Jolla CA).

### Ethics statement

This study was approved by Osaka City University Hospital Certified Review Board (Permission number: CRB5180003) and carried out according to the guidelines of the committee. Written informed consent was obtained from all individual participants included in the study. And this study has been conducted according to the principles of the declaration of Helsinki. Clinical sequencing and data handling was performed under Japan’s personal information protection laws.

## Results

### The clinicopathological features of patients with refractory tumor

A total of 39 patients with advanced solid tumor, including primary unknown site, were enrolled in clinical sequencings using gene panel testing. Among these 39 patients, we identified 36 patients with formalin- fixed paraffin- embedded (FFPE) tumor tissues samples which were available for sequencing with NGS, whereas the remaining 3 patients were excluded from clinical sequencing due to lack of quality FFPE samples.

**Fig 2:**
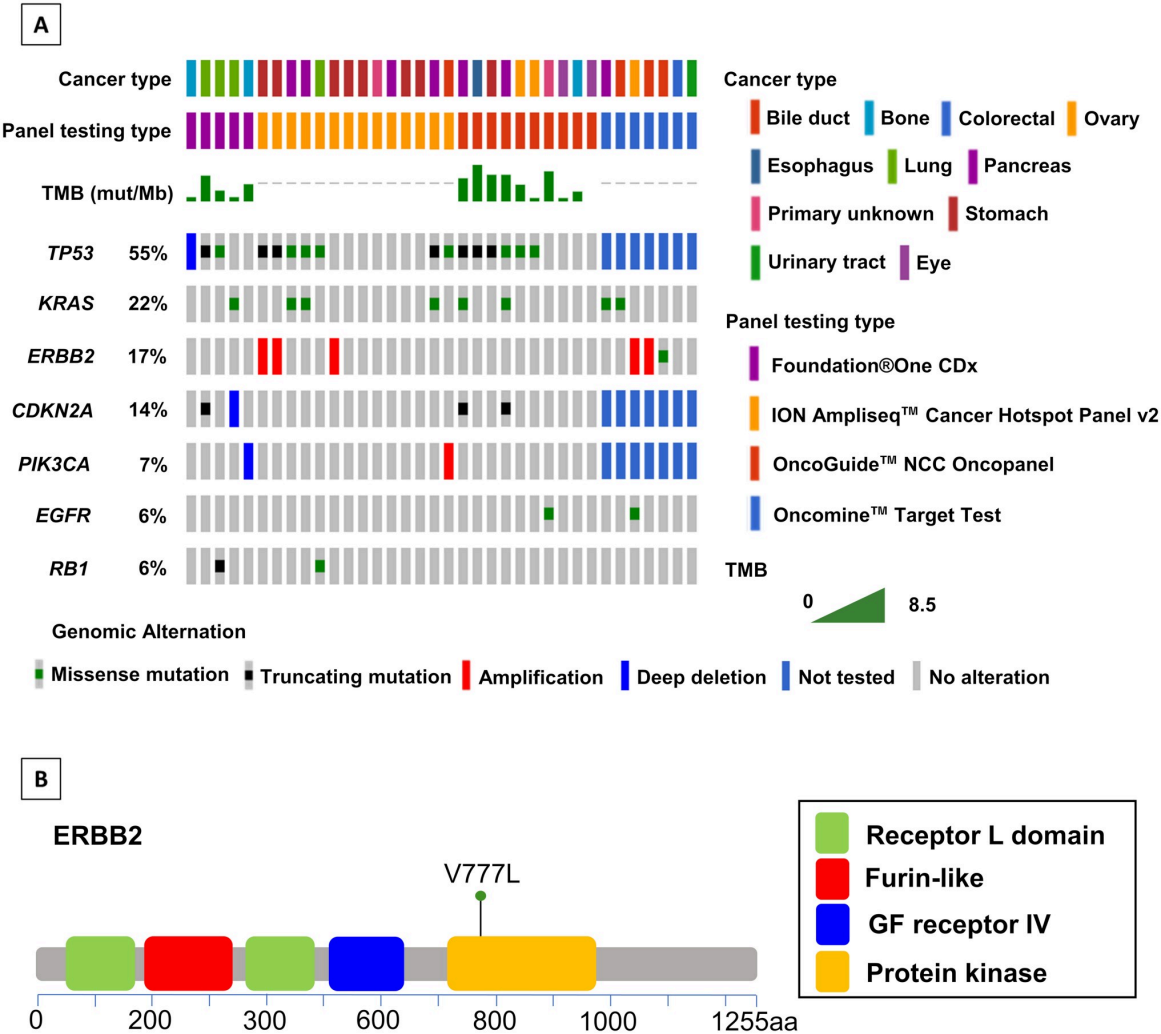
Pathogenic gene alterations in the 36 patients enrolled in clinical sequencing. **A**, Among 36 patients, a total of 7 mutated genes that harbored a point mutation or copy number variation were detected in at least 2 patients. Genome signatures such as the TMB are also described in the list. **B**, Missense mutation, a mutation in codon 777 (Valine → Leucine), was located on the protein kinase position on a liner protein of *ERBB2*.

Of the 36 patients, ten were clinically examined with the NCC Oncopanel, five with the FoundationOne^®^ CDx, 14 with the ION Ampliseq^™^ Cancer Hotspot Panel v2, and seven with the Oncomine^™^ Target Test (Table 1, Fig 2A). Table 1 summarizes the clinical characteristics of the 36 patients. Their median age was 67 years. Among the 36 patients, we identified 21 patients with gastrointestinal (GI) cancer (n = 10) or pancreato-biliary (n = 11) cancer; The remaining fifteen patients had different tumor types, including ovarian cancer (n = 3), lung cancer (n = 4), and bone tumor (n = 3). The rate of DNA extraction from the formalin-fixed paraffin-embedded (FFPE) primary lesion samples was 53% (19/36), and an FFPE sample archived within 3 years from the biopsy was obtained in 30 cases. A successful NGS assay was achieved for all of the patients in this clinical sequencing.

**Table 1:** Patient demographics in clinical sequencing.

Variables		Number of patients	(%)
Age	Mean [range] years old	67 [56–73]^†^	
Sex			
	Female	14	(39)
	Male	22	(61)
Type of Panel testing			
	NCC Oncopanel	10	(28)
	FoundationOne CDx	5	(14)
	Oncomine Target System	7	(19)
	ION Ampliseq hotspot panel v2	14	(39)
Tumor type			
	Bile duct	4	(11)
	Bone	3	(8)
	Colorectal	1	(3)
	Esophagus	1	(3)
	Lung	4	(11)
	Ovary	3	(8)
	Pancreas	7	(19)
	Primary unknown	2	(6)
	Stomach	8	(22)
	Urinary Tract	1	(3)
	Eye	2	(6)
Specimen lesion			
	Primary lesion	19	(53)
	Metastasis lesion	12	(36)
	Unknown	4	(11)
Time from extaction of specimen to sequencing			
	3 years <	6	(17)
	≤3 years	30	(83)
Time from consent to result	Mean [range] Days	47[36–51]	

^†^ Values are median [interquartile range].

### Genomic profile of patients using gene panel

The target resequencing of NCC Oncopanel covered 80%–86% of the coding regions of the genes, while the other panel testing did not disclose the percentage of covered regions. A median read depth within the region of interest in patients using NCC Oncopanel, FoundationOne^®^ CDx, Oncomine^™^ Target Test, and ION Ampliseq^™^ hotspot panel v2 were 1513, 925, 1719, and 10318, respectively (S5 and S6 Tables). Fig 2A provides a list of the frequent mutated genes identified with the use of cBioportal [17,18]. Pathogenic genetic alterations were detected in 28 (78%) of the 36 patients; the panel test showed no mutation in the other eight patients (S6 Table). The most common mutation was *TP53* (55%), followed by *KRAS* (22%). The highest frequency of gene amplification was *ERBB2* (17%) in the clinical sequencing, whereas other amplifications was observed in one patient (Fig 2A). For the two patients who underwent both the ION panel and the Oncomine test, no difference was observed in cancer treatment according to results of the panel testing. There were no patients with a high tumor mutational burden (TMB) in this clinical sequencing, and a germline mutation which contributes to hereditary disease was identified in 1/36 (3%) patients. Rearrangement was not observed in any of these patients with solid tumors.

### Influence on cancer therapy according to result of panel test

The panel results revealed actionable mutations that might be a therapeutic target in fourteen of the 30 patients with pathogenic genetic alterations (Table 2). Five of the fourteen patients had received targeted therapy as a standard therapy, and the other nine patients were identified as candidates for novel molecular-targeted therapy based on their actionable gene alterations. One of these nine patients had cholangiocarcinoma that harbored *ERBB2* missense mutation (V777L) (Fig 2B), which was the therapeutic target for patients with different types of tumors as reported [19,20]. However, treatment with *ERBB2* inhibitors, including neratinib, was approved for solid tumors with *ERBB2* amplification but not for solid tumors with *ERBB2* variant by the Osaka City University Hospital Certified Review Board (certification no. CRB5180003). Another three of these nine patients did not receive genotype-matched therapy because of their poor performance status when they were considered for enrollment in a clinical trial or off label use. The remaining two patients were not followed up because of their transfer to another hospital after testing. Consequently, two (6%) of the patients were candidates for novel targeted therapy after the genetic tests in this study and only one patient has since received subsequent targeted therapy 3 months after sequencing.

**Table 2:** 14 candidates for molecular-targeted therapy based on their actionable gene alterations.

Age	Sex	Tumor type	Gene	Genetic Alternation	Drug	Received target Therapy
79	F	Stomach	*ERBB2*	Amplification	Approved Drug	Yes(standard therapy)
84	F	Stomach	*ERBB2*	Amplification	Approved Drug	Yes(standard therapy)
75	M	Stomach	*ERBB2*	Amplification	Approved Drug	Yes(standard therapy)
56	M	Lung	*EGFR*	Missense(L861Q,T790M)	Approved Drug	Yes(standard therapy)
67	F	Bile Duct	*ERBB2*	Missense (V777L)	Off-label Use	No(refusal of off-label use)
68	F	Ovary	*ERBB2*	Amplification	Investigational Drug	No(poor performance status; PS2*)
72	M	Bile Duct	*ERBB2*	Amplification	Investigational Drug	No(poor performance status; PS2*)
51	M	Lung	*EGFR*	Insertion	Investigational Drug	Unknown
			*CDK4*	Amplification	Off-label Use	Unknown
61	F	Primary Unknown Site	*PIK3CA*	Missense (E777K)	Overseas Approved Drug Off-label Use	Unknown
56	F	Ovary	*BRCA2*	Truncation	Approved Drug	Yes(standard therapy)
75	M	Esophagus	*PTCH1*	Truncation	Overseas Approved Drug	No (not approved in our home country)
16	F	Bone	*PDGFRA*	Amplification	Off-label Use	No(poor performance status; PS2*)
60	F	Lung	*CCND1*	Amplification	Investigational Drug	Not yet(novel therapy)
24	F	Bone	*TSC2*	Deletion	Off-label Use	Yes(clinical trial)

* PS means the scale of performance status developed by the Eastern Cooperative Oncology Group.

### Short survival

**Fig 3:**
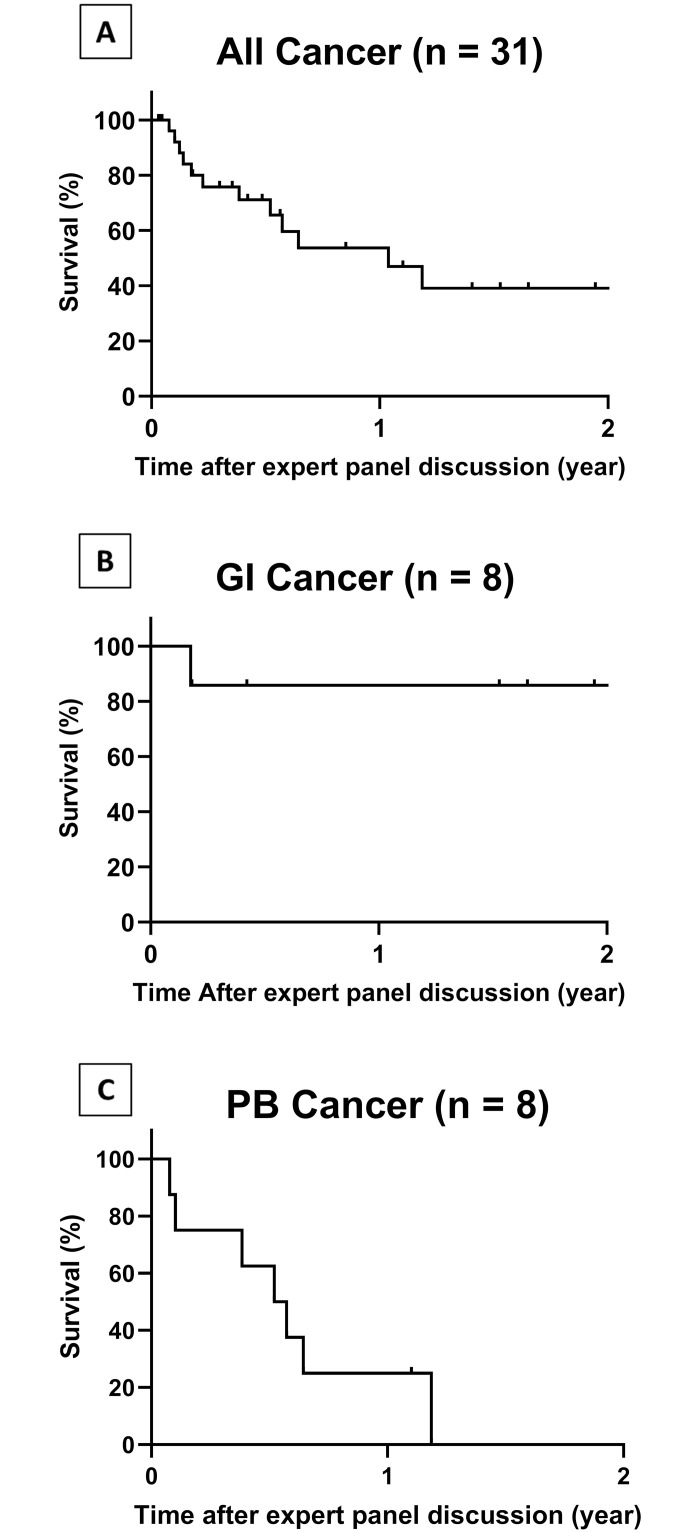
Survival curve of post-panel testing cases. **A**, Half of 31 patients died within 1 year after the panel testing. **B**, The 1-year survival rate of the patients with GI cancer was 87.5%. **C**, The 1-year survival rate of the patients with pancreato-biliary was 25%. PB means pancreato-biliary.

The median period between the date of consent for undergoing the NGS and the date when the NGS results were received was 47 days (Table 1). 5 patients died before their results were returned to the clinician (Table 3). Fig 3A illustrates the survival rate of the 31 patients with advanced solid tumors (the 5 patients who died are excluded). The median period of survival after the expert panel discussion was approx. 1 year. Most of the patients with pancreato-biliary cancer died within 1 year after the expert panel discussion, whereas most of patients with GI cancer were alive >1 year later despite being refractory to standard therapy (Fig 3B and 3C).

**Table 3:** 5 patients who could not receive their panel testing results during their alive.

Age	Sex	Tumor type	Time from sample registration to death (day)	Time from diagnosis to death (year)
72	F	Colorectal	57	9.4
56	M	Bile duct	31	1.5
79	F	Stomach	1	1.2
70	F	Pancreas	27	0.9
70	M	Pancreas	33	1.1

## Discussion

Gene panel testing to detect actionable genomic alterations for therapeutic purpose could test a number of cancer associated genes in a single round by advance in NGS technology, which might replace existing clinical management, including molecular testing of single gene (*e*.*g*., *BRAF*, *ALK*) or composite genetic signature (*e*.*g*., mismatch repair), in the view of cost effectiveness and time-consuming [21]. On the other hand, the NGS test was not basically indicated for cancers at early stage [22]. In this study, we performed practical clinical sequencing for patients with refractory solid tumor using four types of gene panel testing by NGS. Differences were observed among the four panels in the number of actionable genes and the number of cancer-associated genes (Fig 1). It has been demonstrated that the rate of patients who received a subsequent therapy based on actionable gene alteration was not significantly different among several types of panel testing [6,8,10,11]. These findings might suggest that the actionable genes to be detected are limited to major cancer-associated genes, such as *ERBB2* and *EGFR*. However, the number of candidates for actionable genes has been increasing, as has the number of molecular-targeted therapeutics. In fact, a number of novel molecular-targeted therapeutics were approved by the FDA during the current clinical sequencing (https://www.fda.gov/Drugs). It could thus provide a great impact on future cancer treatment to evaluate as many cancer-associated genes as possible, using larger-panel testing, *i*.*e*., whole exome sequencing.

In the present clinical sequencing, 28 of the 36 patients had pathogenic genetic alterations, and fourteen of those 36 patients (38.9%) had an actionable gene for a therapeutic target, which was concordant with the results of earlier studies (ranging from 36.7% to 59.4%) [6,7,10,11]. However, only one of the present patients underwent a novel targeted therapy after their clinical sequencing, whereas over 10% of the patients could receive novel therapy in previous studies [6–8,10,11]. The lack of effective therapeutic agents based on the genomic profiling was definitely one of the reasons for the current results, in addition, there are two more important points to be noted about the present results. First, the most common reason for not undergoing targeted therapy was clinical deterioration during the turnaround time from consent to genetic results (approx. 7 weeks in this patient series). Several reports demonstrated that 6% to 27% of patients fail to receive genome-matched therapy after panel testing because of declining performance status [6,7]. Futhermore, the average of 47 days for NGS results in this study seems to be longer, in compared to previous reports [6,10], while a 47-day delay is typical for clinical settings in Japan [12,13]. One of the reasons for the delay for returning results to patients might be due to the additional timeline of expert panel, which is usually held 3 weeks after sequencing in Japan. Some patients with actionable mutations were unable to receive novel molecular-targeted therapy due to poor performance status by the delay for returning results. An additional challenge of shortening the period from sequencing to expert panel will be necessary. Second, there were two patients in this study who were unable to enroll in a genome-matched trial due to the complexity of gaining entry to a clinical trial even in Japan, and clinical deterioration occurred during the delay. To address these problems, Chantrill et al. suggested that establishing a dedicated multidisciplinary team (including a molecular pathologist responsible for extracting high-quality samples from specimens, and a molecular tumor board meeting member) is necessary to generate a quick turnaround time [23] Regarding the complexity of gaining access to a clinical trial, the utility of a virtual clinical trial—which is designed as a remote trial to evaluate the clinical data of patients via the Internet without hospital visits—has been proposed [24,25]. This challenge seemed like a futuristic idea but today, it is a vital tool for patients and doctors to use telemedicine [26]. This online method might allow clinical patients to reduce their activities for a clinical trial, including the trial entry and travel to a hospital. A combination of these tools might help make clinical sequencing more widely available and accessible for patients hoping to undergo treatment based on genomic profiling.

Another unexpected finding of the present study is that unapproved drug for actionable mutated gene such as *KRAS* and *ERBB2*, was limited to be available, which was different from the result of previous studies [8,11,12]. Notably, a molecular-targeted drug approved for cancer with a specific gene alteration might be active or inactive in patients with different types of tumors. Moreover, it may be difficult to assess comparisons of unapproved drug or investigational drug uses in studies conducted in different countries or regions [27]. Thus, establishing the evidence of utility for off-label use across the world (*i*.*e*., Basket trials) might make it easier to provide a novel therapy for patients with refractory cancer while the practice of unapproved drug use should nevertheless be regulated based on greater security and robust clinical guidance.

In the present study, 14% of the patients died during the turnaround time, and the patients who died within 4 months after registration of panel testing accounted for 30% of the total patients in this clinical sequencing (Table 3, Fig 3A). A low frequency of actionable genes and poor prognoses were observed in the patients with pancreato-biliary cancer, whereas the patients with GI cancer could be treated with an alternative molecular-targeted drug (*e*.*g*., ramucirumab and nivolumab) for standard therapy. These findings will contribute to the identification of the appropriate timing and clinical stage at which clinical sequencing should be performed according to tumor type. Considering that some patients with refractory solid tumor (such as rare tumors and pancreato-biliary cancer) experience impressive responses to targeted therapy following genetic tests [11,12,28], we recommend that clinical sequencing for those patients be performed at an earlier stage (*i*.*e*., at the time of tumor diagnosis or prior to standard chemotherapy).

## Conclusions

The results of the current study indicate that clinical sequencing might be useful for the detection of pathogenic alterations and management of cancer treatment. However, the presence of actionable gene mutation is not necessarily associated with subsequent therapies in patients with solid tumor refractory to standard therapy. In fact, only 3% of the patients received a novel targeted therapy based on this clinical sequencing, and thus further explorations of the optimal timing of clinical sequencing and a consensus about off-label use, as well as a novel molecular target drug development, could help cancer patients benefit from clinical sequencing.

## Supporting information

S1 Table Details of all gene lists in FoundationOne^®^CDx. (XLSX)

S2 Table Details of all gene lists in OncoGuide^™^ NCC Oncopanel. (XLSX)

S3 Table Details of all gene lists in ION Ampliseq^™^ Cancer Hotspot Panel v2. (XLSX)

S4 Table Details of all gene lists in Oncomine^™^ Targt Test. (XLSX)

S5 Table Sequence run summary in 36 patients. (XLSX)

S6 Table A list of all pathogenic genes in 36 patients. (XLSX)

S1 File Molecular profiling assays method. (DOCX)
