# Supplementary material for: Clinical benefit for clinical sequencing using cancer panel testing
Source: PLoS One. 2021 Feb 26;16(2):e0247090. doi: 10.1371/journal.pone.0247090 (PMC7909652; doi:10.1371/journal.pone.0247090)
Supplement: S1 File — (DOCX) [file pone.0247090.s007.docx]

**Molecular profiling assays method**

**OncoGuide™ NCC Oncopanel**

The Innovation Genome Center of RIKEN GENESIS Co., Ltd performed sequencing on the NextSeq 550Dx (Illumina, San Diego, CA). Data analysis was performed using OncoGuide™ NCC OncoPanel Analysis Program ver.1.02-00., which is original NGS data analysis pipeline from the Sysmex Corporation and consists of Burrows-Wheeler Aligner (alignment tool), cisCall-7.1.7 (variant caller) and cisAnnotate-1.1.4 (annotation tool).

**FoundationOne^®^ CDx**

FoundationOne^®^ CDx was only performed and analyzed at the Foundtation Medicine, Inc., which undisclosed molecular profiling assay protocol, including sequencing and bioinformatics method.

**Oncomine^TM^ Target Test**

Sequencing was performed by Ion PGM Dx Sequencer (Thermo Fisher Scientific, Waltham, MA) at Osaka University hospital. Analysis of sequencing raw data was performed by Torrent Suite v.5.4 software program (Thermo Fisher Scientific, Waltham, MA). Tvc 4.50-0 (SNAPSHOT) and Oncomine Reporter (Thermo Fisher Scientific, Waltham, MA) were used for variant calling and genome annotation, respectively.

**Ion Ampliseq**™ **cancer hotspot panel v2**

Department of genome biology at Kindai University performed sequencing on the Ion PGM or Ion Proton sequencer (Thermo Fisher Scientific, Waltham, MA). Sequence alignment and base calling used Torrent Suite v.5.2 software program, followed by variant calling using Variant Caller plugin v.5.2 (Thermo Fisher Scientific, Waltham, MA)., and sequencing coverage analysis using Coverage Analysis plugin v5.2 (Thermo Fisher Scientific, Waltham, MA).
